# Passive Synaptic Normalization and Input Synchrony-Dependent Amplification of Cortical Feedback in Thalamocortical Neuron Dendrites

**DOI:** 10.1523/JNEUROSCI.3836-15.2016

**Published:** 2016-03-30

**Authors:** William M. Connelly, Vincenzo Crunelli, Adam C. Errington

**Affiliations:** ^1^Neuroscience Division, School of Biosciences, Cardiff University, Cardiff CF10 3AX, United Kingdom,; ^2^Department of Physiology and Biochemistry, University of Malta, Msida MSD 2080, Malta,; ^3^Neuroscience and Mental Health Research Institute, School of Medicine, Cardiff University, Cardiff CF24 4HQ, United Kingdom, and; ^4^Eccles Institute of Neuroscience, The John Curtin School of Medical Research, Australian National University, Canberra City, Australian Capital Territory 2600, Australia

**Keywords:** dendritic integration, NMDA receptor, passive normalization, T-type calcium channel, thalamocortical

## Abstract

Thalamocortical neurons have thousands of synaptic connections from layer VI corticothalamic neurons distributed across their dendritic trees. Although corticothalamic synapses provide significant excitatory input, it remains unknown how different spatial and temporal input patterns are integrated by thalamocortical neurons. Using dendritic recording, 2-photon glutamate uncaging, and computational modeling, we investigated how rat dorsal lateral geniculate nucleus thalamocortical neurons integrate excitatory corticothalamic feedback. We find that unitary corticothalamic inputs produce small somatic EPSPs whose amplitudes are passively normalized and virtually independent of the site of origin within the dendritic tree. Furthermore, uncaging of MNI glutamate reveals that thalamocortical neurons have postsynaptic voltage-dependent mechanisms that can amplify integrated corticothalamic input. These mechanisms, involving NMDA receptors and T-type Ca^2+^ channels, require temporally synchronous synaptic activation but not spatially coincident input patterns. In hyperpolarized thalamocortical neurons, T-type Ca^2+^ channels produce nonlinear amplification of temporally synchronous inputs, whereas asynchronous inputs are not amplified. At depolarized potentials, the input–output function for synchronous synaptic input is linear but shows enhanced gain due to activity-dependent recruitment of NMDA receptors. Computer simulations reveal that EPSP amplification by T-type Ca^2+^ channels and NMDA receptors occurs when synaptic inputs are either clustered onto individual dendrites or when they are distributed throughout the dendritic tree. Consequently, postsynaptic EPSP amplification mechanisms limit the “modulatory” effects of corticothalamic synaptic inputs on thalamocortical neuron membrane potential and allow these synapses to act as synchrony-dependent “drivers” of thalamocortical neuron firing. These complex thalamocortical input–output transformations significantly increase the influence of corticothalamic feedback on sensory information transfer.

**SIGNIFICANCE STATEMENT** Neurons in first-order thalamic nuclei transmit sensory information from the periphery to the cortex. However, the numerically dominant synaptic input to thalamocortical neurons comes from the cortex, which provides a strong, activity-dependent modulatory feedback influence on information flow through the thalamus. Here, we reveal how individual quantal-sized corticothalamic EPSPs propagate within thalamocortical neuron dendrites and how different spatial and temporal input patterns are integrated by these cells. We find that thalamocortical neurons have voltage- and synchrony-dependent postsynaptic mechanisms, involving NMDA receptors and T-type Ca^2+^ channels that allow nonlinear amplification of integrated corticothalamic EPSPs. These mechanisms significantly increase the responsiveness of thalamocortical neurons to cortical excitatory input and broaden the “modulatory” influence exerted by corticothalamic synapses.

## Introduction

Thalamocortical neurons (TCs) transfer peripheral information to the cortex by relaying signals they receive from sparse, but powerful, proximal dendrite targeting sensory synapses (Sherman, 2007). However, the numerically dominant synaptic input to TC neurons comes from several thousand corticothalamic (CT) synapses (Liu et al., 1995; Van Horn et al., 2000), distributed widely across their dendritic tree, that provide substantial top-down moment-by-moment feedback modulation of sensory information flow (Sillito and Jones, 2002; Cudeiro and Sillito, 2006; Briggs and Usrey, 2008). Indeed, TC neurons are also innervated by local GABAergic interneurons and thalamic reticular nucleus (TRN) neurons, which themselves receive direct cortical input, meaning that 60%–70% of synapses on TC neurons are influenced directly or indirectly by cortical activity (Wolfart et al., 2005). Nonetheless, the precise function of CT feedback remains uncertain.

A significant barrier to understanding the full function of CT feedback is the lack of information regarding how specific input patterns are integrated by TC (and TRN) neurons. Critically, previous studies using electrical or optogenetic stimulation have examined only the extreme ends of the temporal input range. As such, the effects of activating individual quantal-sized EPSPs have been explored (Golshani et al., 2001; Granseth and Lindström, 2003), as have responses evoked by synchronous stimulation of multiple CT inputs both *in vitro* and *in vivo* (Turner and Salt, 1998; Cruikshank et al., 2010; Jurgens et al., 2012; Augustinaite et al., 2014; Mease et al., 2014; Crandall et al., 2015). However, as well as providing little information on the effects that temporal input pattern has on CT responses, these studies do not answer questions regarding the consequences of the spatial distribution of inputs. Considering that in some neurons, as a consequence of dendritic filtering, distal EPSPs exert a smaller effect on somatic output than EPSPs with a more proximal origin (Rall, 1967; Williams and Stuart, 2002), understanding how electrotonic and morphological properties of TC neuron dendrites influence CT EPSPs is important. Recently, we revealed using dendritic recording that low threshold (LT) spiking neurons, including TC neurons, have electrotonic properties that may negate the effects of dendritic filtering on distal synaptic inputs (Connelly et al., 2015). Although these findings support previous computational modeling studies (Briska et al., 2003; Perreault and Raastad, 2006; Lajeunesse et al., 2013), this hypothesis remains untested experimentally; thus, whether distal CT EPSPs exert as powerful an influence over somatic membrane potential as more proximal inputs remains unknown. Furthermore, local integration of synaptic inputs can result in significant nonlinearity in neuronal input–output (I-O) functions (i.e., dendritic Ca^2+^ or NMDA spikes) depending upon the spatiotemporal pattern of input (Losonczy and Magee, 2006; Branco et al., 2011; Abrahamsson et al., 2012). Although dynamic clamp studies have explored effects of cortically generated stochastic noise (Wolfart et al., 2005; Deleuze et al., 2012; Béhuret et al., 2013) on TC neuron signaling, the effects that specific spatial and temporal input patterns have on TC neurons during state-dependent “tonic” and “burst” firing modes remain unknown. For example, are CT inputs spatially clustered onto individual dendritic branches integrated in a nonlinear manner, and does this differ from CT inputs widely distributed in space across the dendritic tree?

To answer these questions, we have taken advantage of the temporal and spatial precision of 2-photon glutamate uncaging to investigate the responsiveness of TC neurons to patterned activation of CT synaptic inputs. Combining experimental findings with computational modeling, we find that postsynaptic mechanisms, requiring T-type Ca^2+^ channels and NMDA receptors, significantly amplify CT input to TC neurons. This amplification relies on the temporal synchrony of synaptic input, is influenced by the spatial location of synapses within the dendritic tree, and broadens the modulatory influence CT feedback can exert upon TC neuron signaling.

## Materials and Methods

### 

#### 

##### Brain slice preparation and dendritic patch-clamp recording.

Coronal slices (300 μm) containing the dorsal lateral geniculate nucleus were prepared from postnatal day 20–25 Wistar rats of either sex deeply anesthetized using isoflurane as previously described (Errington et al., 2010; Connelly et al., 2015) with approval of the Cardiff University Research Ethics Committee and in accordance with the Home Office Animals (Scientific Procedures) Act 1986, United Kingdom. For recording, slices were transferred to a submersion chamber continuously perfused with warmed (33°C-4°C) aCSF (mm) as follows: 125 NaCl, 2.5 KCl, 2 CaCl_2_, 1 MgCl_2_, 1.25 NaH_2_PO_4_, 25 NaHCO_3_, and 25 d-glucose (305 mOsm) at a flow rate of 2.5–3 ml/min. Somatic whole-cell patch-clamp recordings were made from TC neurons (visually identified by infrared gradient contrast video microscopy) using a Multiclamp 700B amplifier (Molecular Devices) and pipettes with resistances of 4–6 mΩ when filled with internal solution containing (in mm) the following: 130 K-gluconate, 20 KCl, 10 HEPES, 0.16 EGTA, 2 Mg-ATP, 2 Na_2_-ATP, 0.3 Na_2_-GTP, pH 7.3 (295 mOsm) and supplemented with 50 μm Alexa-594 (Invitrogen). Two-photon excitation fluorescence targeted dendritic recordings were performed as described by Connelly et al. (2015). Briefly, 2-photon fluorescence microscopy, using a Prairie Ultima (Prairie Technologies) microscope and titanium:sapphire pulsed laser (Chameleon Ultra II; Coherent) tuned to λ = 810 nm, was combined with IR-scanning gradient contrast to make targeted dendritic patch-clamp recordings. To record from thin dendrites, high resistance recording electrodes (32–40 mΩ), made from borosilicate glass capillaries (BF200-100-10, Sutter Instruments), were used. Dendritic and somatic bridge balance and pipette capacitance neutralization were carefully adjusted and monitored throughout experiments by application of brief low amplitude current steps (100 Hz, 10–30 pA). Electrophysiological data were sampled at 20–50 kHz and filtered at 6 kHz. Somatic series resistance at the start of experiments was between 9 and 15 mΩ and varied ≤20% during recordings.

##### Variance-mean analysis and synaptic current injection.

The theory of estimating quantal parameters using variance-mean analysis has been extensively discussed by others (Silver et al., 1998; Reid and Clements, 1999; Clements and Silver, 2000). We used the simple form of this analysis to obtain approximate quantal amplitudes for corticothalamic EPSC. Briefly, for an average synaptic current (I):

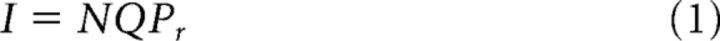
 where *N* is the number of release sites, *Q* is the amplitude of a single quantal event, and *P_r_* is the probability of release of a neurotransmitter quantum from a release site. The variance of the amplitude of the synaptic current (σ^2^) is given by the following:

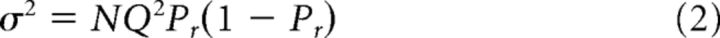
 and eliminating *P_r_* between these two equations (Eqs. 1, 2) gives the following:

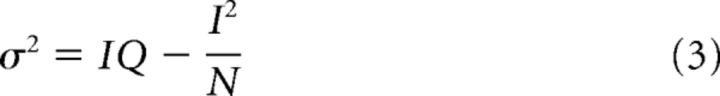
 Release probability can then be calculated by the following:

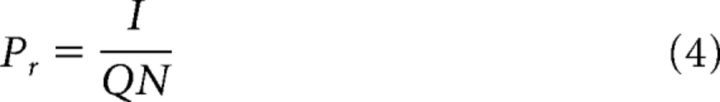
 To account for variability in quantal size at individual release sites a correcting factor, the intrasite coefficient of variation (CV_1_^2^), is often used. Calculating this parameter in intact slices presents several serious difficulties; as such, we have not used this in our estimations. Nonetheless, adding CV_1_^2^ to Equation 3 gives the following:

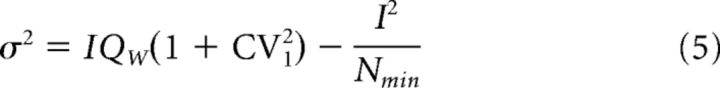
 Based upon the CV_1_^2^ estimated by Ikeda et al. (2008) in isolated hippocampal cultures, addition of this factor would only reduce our estimate of quantal size from 10.1 ± 0.9 pA to 8.7 ± 0.5 pA (*p* > 0.05).

In dendritic recordings, local spontaneous unitary EPSPs (sEPSPs) recorded at the dendritic electrode were selected according to rise time (≤0.5 ms). Amplitudes of dendritic and somatic sEPSPs were measured from averages of all detected events, and attenuation was calculated as the ratio of the peak amplitude of the dendritic sEPSP to that recorded at the soma. Injected artificial EPSPs (aEPSPs) were evoked by injection of EPSC-like currents (aEPSCs) through the dendritic recording electrode whose amplitude and kinetics were determined by the results obtained from quantal analysis.

##### 2-photon MNI-glutamate uncaging.

MNI-caged l-glutamate (10 mm, Tocris Bioscience) was dissolved in HEPES-buffered aCSF and locally puffed over the surface of the brain slice via a low resistance patch pipette (tip diameter ∼5–7 μm). In pilot experiments, Alexa-488 (20 μm) was included to determine radial spread of the caged compound and guide positioning of the applicator pipette to achieve uniform concentration near the dendrites chosen for uncaging experiments. Intact distal dendrites (>100 μm from the soma), where segments ≥30 μm in length were observed in a narrow *Z*-plane near the surface of the slice (∼30 μm depth), were selected for uncaging experiments. Two-photon uncaging was performed using a titanium:sapphire laser (Chameleon Ultra II, Coherent) tuned to λ = 720 nm and an uncaging dwell time of 0.5 ms per spot. The intensity of the uncaging laser beam and the duration of uncaging pulses were controlled by electro-optical modulators (Conoptics). Up to 16 uncaging spots were chosen with a minimum interspot distance of 2 μm. Multisite patterned uncaging was achieved using a galvanometer-based scanning system (Prairie Technologies) by rapid (minimum move time 0.1 ms) point-to-point movement of the laser beam. Laser power was minimized to prevent photodamage (<10 mW at slice surface).

**Figure 1.**
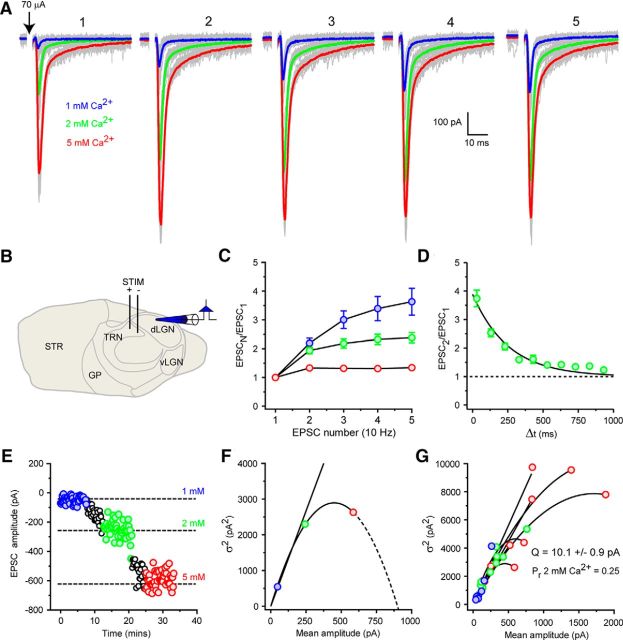
Variance-mean analysis of CT EPSC quantal size. ***A***, Trains (10 Hz) of evoked CT EPSCs in a representative dLGN TC neuron under varying extracellular Ca^2+^ concentrations. Blue traces represent mean EPSC 1 mm Ca^2+^. Green traces represent mean EPSC 2 mm Ca^2+^. Red traces represent mean EPSC 5 mm Ca^2+^. Gray traces represent individual EPSCs. ***B***, Schematic of slice preparation showing placement of stimulating and recording electrodes. ***C***, EPSC frequency-dependent facilitation typical of CT EPSCs. ***D***, Time-dependent recovery of CT EPSCs from facilitation (*n* = 6). ***E***, A typical experiment (traces shown in ***A***) used for VM analysis of quantal size. Same colors as in ***A***. At each Ca^2+^ concentration, 40–50 evoked EPSCs were collected. The increase in mean EPSC amplitude is accompanied by an increase of the variance of the response with increasing Ca^2+^ concentration. ***F***, Plot of mean EPSC amplitude versus EPSC amplitude variance (σ^2^) for 50 trials at different Ca^2+^ concentrations. Same colors as in ***A***. A second-order polynomial (quadratic) fit of the data yields a quantal size (Q, initial slope) of 10.7 ± 2.0 pA. ***G***, Overlaid individual fits to data from 11 dLGN TC neurons. Mean values for Q and release probability in 2 mm Ca^2+^ (P_r_ 2 mm Ca^2+^) are shown.

Distal dendrites of thalamocortical neurons are aspiny but receive dense CT input with synapses forming at average distances of ∼1–2 μm (Wilson et al., 1984; Sherman and Guillery, 1996). In the absence of spines, which present obvious targets for uncaging, it was necessary to place our uncaging spots near to the distal dendritic shaft. To activate individual synapses with each uncaging input, an approximation of the “effective uncaging range” was calculated. First, to minimize the point spread function of our 2-photon microscope and reduce the 2-photon uncaging volume, we chose an objective lens with a high numerical aperture (Olympus LUMPlan FL/N, 60×, 1.0 NA) and ensured correct filling of the back aperture. The FWHM amplitude of the lateral (0.41 μm, *n* = 4 beads) (see Fig. 4*B*) and axial imaging (1.6 μm, *n* = 4 beads) (see Fig. 4*C*) point spread function were experimentally measured using subdiffraction limit PS-speck (0.17 μm) fluorescent microspheres (Invitrogen) and found to be close to those estimated theoretically for this lens at λ = 810 nm (lateral: 0.31 μm; axial: 1.1 μm) (see Fig. 4*A–C*) (Zipfel et al., 2003). This approximates to a 2-photon excitation volume of ∼0.7 fL (see Fig. 4*A*). Subsequently, whole-cell patch-clamp recordings were performed on hippocampal CA1 pyramidal neurons filled with Alexa-594 (50 μm) and spiny regions of basal dendrites chosen for uncaging (see Fig. 1*D*). Uncaging was initially performed at several chosen spines (see Fig. 4*D*, red circles) using an uncaging dwell time of 0.5 ms and laser power adjusted to give uncaging-evoked EPSPs (uEPSPs) typical of these cells. Subsequently, an individual spine was chosen (see Fig. 4*D*, green circles) and the uncaging spot moved progressively toward or away (1 μm increments) from the optimal uncaging site (spine head) (uEPSP: 1.14 ± 0.17 mV, *n* = 5). The amplitudes of uEPSPs at each distance from the spine head were plotted and fit with a Gaussian function yielding an FWHM of 1.32 μm (*n* = 5) (see Fig. 4*E*). At 2 μm from the spine head, virtually zero (0.04 ± 0.01 mV, 3.9 ± 1.4% of maximum, *n* = 5) response to uncaged glutamate was observed (*n* = 5) (see Fig. 4*E*). Thus, for uncaging experiments on TC neuron dendrites, we used a minimum separation of 2 μm to minimize overlap of the “effective uncaging range” of each spot. The resultant uEPSPs had amplitudes and shapes similar to synaptically evoked CT EPSPs, although rise times were typically slightly slower. Furthermore, the NMDA-to-AMPA ratio of uncaging evoked EPSCs (uEPSCs) was not significantly different from that of evoked EPSCs at −50 mV (see Fig. 4*F*,*G*). Uncaging at individual spots with an interval of 400 ms was used to obtain the uEPSP response for each site (see Figs. 5*B*, 9*B*), and these were used to calculate the arithmetic sums of events in some experiments. The “noisy baseline” of TC neurons and the relatively small size of uEPSPs meant it was not possible to adequately resolve individual inputs in all dendrites tested. Only neurons where individual uEPSPs could be clearly resolved were used for calculation of the expected summed EPSPs. Single-site uEPSPs are averages of 10–20 trials, and the number of trials was minimized where possible to avoid photodamage to targeted dendrites. Input order was randomly determined in software (Triggersync, Prairie Technologies). The stellate shape of TC neurons typically limited the length of dendrite within a narrow axial (*Z*) range to ∼30 μm; as such, the number of potential uncaging sites was limited due to the spacing considerations described above. Therefore, we adjusted uncaging laser power (<10 mW) to give uEPSPs 2–2.5 times larger than quantal-sized EPSPs (mean uEPSP: 0.37 ± 0.01 mV, *n* = 183 from 12 dendrites). This allowed us to evoke larger uEPSPs and explore a wider range of membrane potential-dependent effects. Data from our computational model confirmed that results obtained using these larger uEPSPs could be completely reproduced when smaller quantal-sized events were used (see Results; Fig. 7*E*,*F*). For pharmacological experiments, drugs were present in the bath solution and the “puffer” application pipette (containing MNI-glutamate) at the concentrations indicated, and all statistical comparisons are between control and drug-treated dendrites from different neurons.

To quantify deviations from linearity in uEPSP summation for synchronous (0.1 ms) and asynchronous (5 ms) input patterns, we compared measured EPSPs evoked by each sequence of inputs (2–16 inputs) with the expected algebraic sums of individual inputs delivered in a temporally distributed fashion (400 ms intervals). In some experiments, a linear regression fit to uEPSPs evoked by low numbers of inputs (2–6 inputs) was used to determine linearity. In experiments where low-threshold Ca^2+^ spikes were observed, data were aligned to the threshold number of inputs at which LTSs were evoked to allow comparisons between different dendritic branches where absolute threshold and size of individual uEPSPs varied. For input delivered at 5 ms intervals, the threshold level for each dendrite was calculated using uncaging pulses at 0.1 ms before commencing asynchronous pattern delivery. For plots of experimental measured versus expected uEPSPs, the mean expected uEPSPs evoked by different input numbers for all dendrites tested are plotted. *x*-axis error bars indicate the SEM for expected uEPSPs and *y*-axis indicates the measured uEPSPs.

##### Computational modeling.

The details of the model TC neuron used in this study are described fully by Connelly et al. (2015). Synaptic currents were modeled by a combination of AMPA/NMDA receptor conductances, using an NMDA receptor model described by Branco et al. (2010). AMPA channels were modeled with NEURON′s *AlphaSynapse*, a conductance-based model that follows an α function. For simulated uncaging experiments, maximum AMPA conductance was 200 pS, whereas maximum NMDA conductance was 1200 pS. This produced an EPSP of ∼350 μV and at −70 mV, an EPSC of 9 pA. At −50 mV, the size of the EPSCs was 7 pA. In the absence of NMDA receptors, the AMPA only EPSC was reduced to 6 pA. Subtraction of AMPA only EPSC from that produced by AMPA/NMDA synapses gave an estimated NMDA receptor-mediated EPSC of 2 pA and an NMDA-to-AMPA ratio of 0.3. To model quantal-sized cortical inputs, AMPA conductance was reduced to 77 pS, and the maximum NMDA conductance was reduced to 462 pS. This kept the NMDA-to-AMPA ratio identical but reduced the resultant EPSP to 150 μV.

For network simulation, the timing of EPSPs was determined by sampling from a Poisson distribution and the spatial location of the synaptic input was chosen randomly from a set of positions on the distal dendrites. Simulations were run repeatedly over a range of mean interevent intervals, and the rate of input was increased until the cell spiked on >50% of trials. Different resting potentials were achieved using current injection at the soma. During the simulation run, EPSP barrages lasted for 1 s, and the depolarization was calculated as the mean membrane potential during the last 500 ms of the barrage in nonspiking trials. Simulations were solved with a fixed time step of 50 μs or using the implicit variable time-step solver CVODE, depending on the nature of the simulation (Cohen and Hindmarsh, 1996).

##### Data analysis and statistics.

Data analysis was performed using pClamp 10 (Molecular Devices), MATLAB (The MathWorks), and Prizm (GraphPad) software. Distance measurements were performed on image stacks collected at the end of recordings using MetaMorph (Molecular Devices) as described by Errington et al. (2010). Statistical testing was by paired/unpaired *t* test or Pearson's *r* test where appropriate. All values are given as mean ± SEM.

## Results

### Location-dependent attenuation and passive normalization of CT synaptic input in TC neuron dendrites

In a previous study, we described the electrotonic properties of TC neuron dendrites, including strong dendrite to soma (D→S) voltage attenuation of steady-state signals (Connelly et al., 2015). These findings not only predict that distal CT EPSPs will be strongly attenuated as they propagate from their dendritic site of origin to the soma but also that their somatic amplitude will be passively normalized (Jaffe and Carnevale, 1999; Briska et al., 2003; Perreault and Raastad, 2006; Lajeunesse et al., 2013). Here, we tested this hypothesis by evoking aEPSPs using injection of EPSC-like currents (aEPSCs) into TC neuron dendrites.

To accurately mimic single CT inputs, we estimated the quantal size (Q) of CT EPSCs using variance-mean analysis. Brain slices were prepared as described by Turner and Salt (1998), and a bipolar stimulating electrode placed into the TRN to stimulate CT afferents (Fig. 1*A*,*B*) in the presence of SR-95531 and CGP-55845 (10 and 5 μm to block GABA_A_ and GABA_B_ receptors, respectively). CT EPSCs, identified by their characteristic paired-pulse facilitation (Granseth et al., 2002), were evoked by trains of 5 stimuli (20–200 μA) at 10 Hz (*n* = 11) (Fig. 1*A*,*C*,*D*). A minimum of 50 synaptic currents were then recorded in each of three external recording solutions containing 1, 2, or 5 mm Ca^2+^ to modify synaptic release probability (P_r_; Fig. 1*A*,*E*). EPSC variance (σ^2^) for the first event in each train and for each Ca^2+^ concentration was then plotted against the mean EPSC amplitude for that condition and fitted with a second-order polynomial (see Materials and Methods; Eq. 3) to yield a value for Q of 10.1 ± 0.9 pA (*n* = 11) (Fig. 1*F*,*G*).

**Figure 2.**
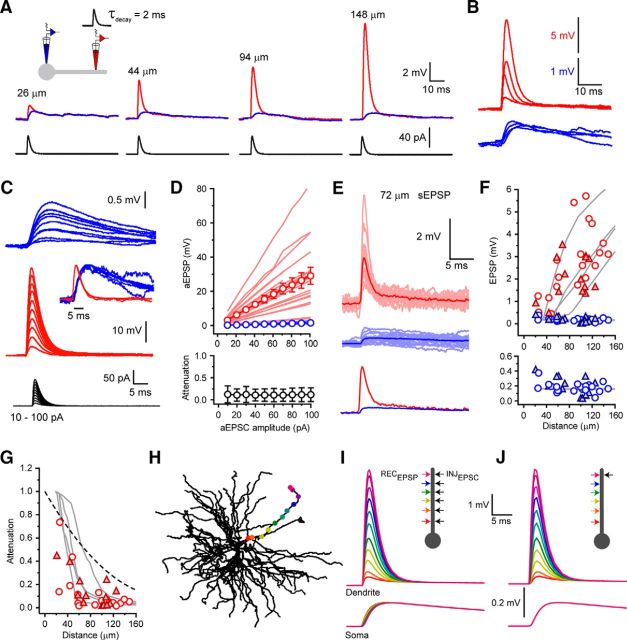
Attenuation and passive normalization of CT EPSPs in TC neuron dendrites. ***A***, Somatic (blue) and dendritic (red) aEPSPs evoked by aEPSC injections (40 pA, black) at increasing distance from the soma in four different TC neurons. ***B***, Overlaid somatic (blue) and dendritic (red) aEPSPs shown in ***A***. Note the marked similarity in somatic aEPSP amplitude, rise time, and decay. ***C***, Traces represent the linear increase in dendritic (red) and somatic (blue) aEPSPs amplitude evoked by increasing-sized aEPSCs (10–100 pA). Inset, Overlaid and peak normalized somatic and dendritic EPSPs have the same rise and decay times. ***D***, Individual (light red) and average (red) dendritic aEPSP amplitude and corresponding individual (light blue) and average (blue) somatic aEPSP amplitude plotted against injected aEPSC size. Bottom graph represents D→S aEPSP attenuation independent of the size of injected aEPSCs. ***E***, Individual (light red) and average (red) dendritic sEPSPs and corresponding individual (light blue) and average (blue) somatic sEPSPs recorded 72 μm from the soma. ***F***, EPSP amplitude versus distance from soma. aEPSPs were evoked by quantal-sized (10 pA) aEPSC injections. Red circles represent dendritic aEPSP. Blue circles represent somatic aEPSP. Red filled triangles represent dendritic sEPSP. Blue filled triangles represent somatic sEPSP. Gray lines indicate data from four model dendrites. Bottom graph represents an expanded view of the peak somatic aEPSP (blue circles) and sEPSP (blue filled triangles) amplitude versus distance of the dendritic current injection from the soma. ***G***, EPSP attenuation in TC neuron dendrites. Red circles represent aEPSPs. Red filled triangles represent sEPSPs. Gray lines indicate model EPSPs. Black dashed line indicates steady-state V_D_→V_S_. ***H***, Model cell showing dendritic locations into which EPSC-like currents shown in ***I*** and ***J*** were injected. ***I***, Input location-dependent model EPSPs. Color-coded dendritic and somatic EPSPs evoked by EPSC injection into the sites shown in ***H***. ***J***, Propagation of distal EPSPs in model dendrites. EPSPs recorded at each location in ***H*** in response to an EPSC injected at the most distal site.

Next, using paired somatodendritic recordings, aEPSCs (minimal amplitude of 10 pA and τ_decay_ = 2 ms) were injected into the dendrites of dorsal LGN (dLGN) neurons (Fig. 2*A*,*C*). First, we found that both dendritic and somatic aEPSP amplitudes were linearly related to injected aEPSC size (10–100 pA) at all dendritic input locations and that D→S aEPSP attenuation did not depend on aEPSC amplitude (Fig. 2*C*,*D*). Consistent with the dendritic input impedance gradient found in TC neurons (Connelly et al., 2015), injection of quantal-sized aEPSCs (10 pA) produced dendritic aEPSPs whose amplitudes increased markedly with distance from the soma (0.45 to 8.20 mV, 3.02 ± 0.43 mV, *n* = 20) (Fig. 2*A*,*B*,*F*). Conversely, the corresponding somatic aEPSPs were significantly smaller (0.04 to 0.38 mV, 0.18 ± 0.01 mV, *n* = 20, *p* < 0.0001, paired *t* test; Fig. 2*A*,*B*,*F*) with dendrite to soma EPSP attenuation (D→S_aEPSP_; aEPSP soma/aEPSP dendrite) ranging from 0.74 (26 μm from soma) to 0.017 (125 μm) (mean D→S_aEPSP_: 0.12 ± 0.04, *n* = 20) (Fig. 2*G*). Equivalent attenuation of injected aEPSPs was observed when neurons were depolarized to V_m_ = −55 mV (*n* = 3, data not shown). Consistent with efficient D→S current transmission and input location independence of somatic voltage phase, we (Connelly et al., 2015) previously reported that both somatic and dendritic aEPSPs had 10%–90% rise times and half-widths that varied little with input location (Fig. 2*A*,*B*). Most importantly, we found somatic aEPSP amplitude was virtually independent of the distance of the dendritic input site from the soma (Fig. 2*A*,*B*,*F*).

We also observed sEPSPs and several lines of evidence allow us to conclude that these are of CT origin. First, local dendritic EPSPs were large and increased in amplitude with increasing distance from the soma. Although this could result from proximal retinal EPSPs propagating into distal dendrites, as a result of efficient soma to dendrite voltage transfer, in this case more uniform sEPSPs amplitudes distal dendrites and larger somatic EPSPs would be expected (because retinal EPSPs are larger than CT EPSPs). Second, dendritic sEPSP amplitudes closely match aEPSPs evoked quantal-sized aEPSCs and show remarkably similar distance-dependent attenuation. Third, somatic sEPSP amplitudes (see below) are not significantly different from those of aEPSPs and similar to those predicted for single quantal events by comparison of electrically evoked CT EPSCs and EPSPs (10 pA EPSC = ∼150–200 μV EPSP; data not shown). Fourth, the selected dendritic EPSPs had rise times of <0.5 ms, suggesting that they originated near the dendritic recording electrode. Because the majority of excitatory synaptic contacts onto the thin dendrites are CT, it is highly likely that the recorded sEPSPs belong to this class of synaptic inputs.

The amplitude of dendritic sEPSPs (0.50–3.55 mV, 2.05 ± 0.25 mV, *n* = 14) were dependent upon input location and remarkably similar to quantal-sized aEPSPs (Fig. 2*E*,*F*), although their decay time constants were slightly less (sEPSP: 2.1 ± 0.3 ms, *n* = 14, aEPSP: 3.2 ± 0.1 ms, *n* = 20, unpaired *t* test, *p* < 0.05). Furthermore, attenuation of sEPSPs was not different from aEPSPs (D→S_sEPSP_: 0.02 to 0.45, 0.14 ± 0.04, *n* = 14, *p* > 0.05; paired *t* test; Fig. 2*G*), resulting in somatic sEPSP amplitudes being independent of input location (0.04 to 0.42 mV, 0.20 ± 0.03 mV, *n* = 14; Fig. 2*F*). These findings were reproduced in simulated dendrites, which also showed location-dependent EPSP attenuation (Fig. 2*G–I*) due to steep distance-dependent increases in dendritic EPSP amplitude and little location dependence of somatic EPSPs (Fig. 2*F*,*I*). Moreover, our computational model shows that, although they are strongly attenuated during dendro-somatic propagation, CT EPSPs show little broadening (Fig. 2*J*). Thus, we conclude that CT synaptic potentials are strongly attenuated as they propagate from their dendritic site of origin but that their amplitude, rise times, and decay times at the soma are normalized to give all inputs equal weight regardless of the spatial input location within the dendritic tree. Moreover, the clear correspondence in location-dependent amplitudes of dendritic aEPSPs, evoked by injection of equal-sized quantal aEPSCs at each site, and the sEPSPs recorded in dendrites (Fig. 2*F*) strongly suggests that this process of normalization is passive, relying on dendritic electrotonic properties, but not on distance-dependent changes in synaptic conductance.

**Figure 3.**
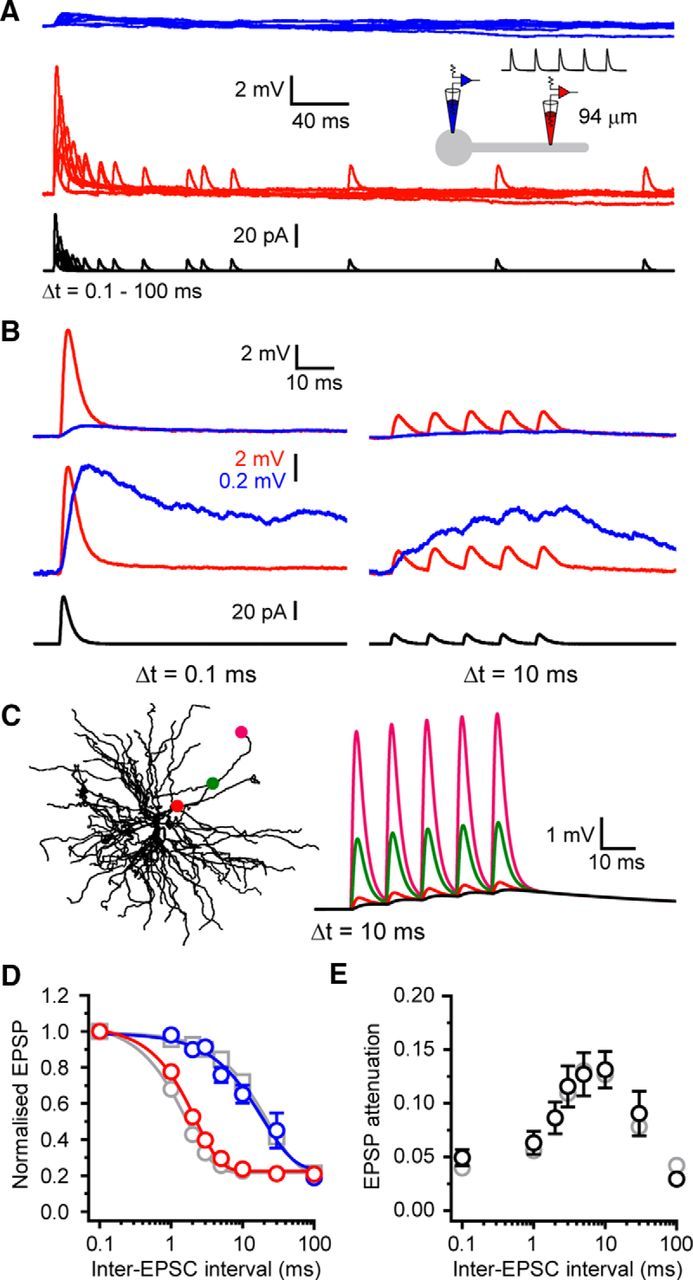
Temporal window for optimal compound CT-EPSP propagation. ***A***, Somatic (blue) and dendritic (red) compound aEPSPs evoked by trains of five injected aEPSCs (black) at varying inter-EPSC intervals (0.1–100 ms). ***B***, Example traces showing somatic and dendritic responses evoked by EPSC trains at 0.1 and 10 ms intervals. Bottom traces represent somatic compound aEPSPs scaled to match the peak dendritic aEPSP at 0.1 ms. The reduced attenuation between aEPSPs with 0.1 and 10 ms intervals for the somatic (blue) versus dendritic (red) recording site is clear. ***C***, Propagation of model compound EPSPs recorded at the locations shown in response to EPSCs evoked at the most distal location (10 ms interval). ***D***, Compound aEPSP amplitude normalized to the amplitude at 0.1 ms intervals versus inter-EPSC interval. Blue circles represent somatic compound aEPSPs. Red circles represent dendritic compound aEPSPs. Gray circles represent model dendritic compound aEPSPs. Gray squares represent model somatic compound aEPSPs. ***E***, Attenuation of compound aEPSPs versus inter-EPSC frequency. Black circles represent experimental data. Gray circles represent model.

The above results relate to attenuation of single CT synaptic inputs. During natural behavior, TC neurons receive input from numerous CT synapses with varying degrees of synchrony. Because we have previously shown that input frequency has a marked effect upon dendrite to soma voltage transfer (Connelly et al., 2015), we tested whether the ability of CT inputs to depolarize the soma was dependent upon input synchrony. To do this, we injected a series of five aEPSCs into dendrites 100–130 μm from the soma with varying inter-EPSC intervals (Δ*t* = 0.1–100 ms, *n* = 9) (see Fig. 9*A*). Compound aEPSPs at all input frequencies were strongly attenuated as they propagated from dendrites to soma (Fig. 3*A*). However, EPSCs with intermediate intervals between 2 and 30 ms (Δ*t* = 10 ms: 0.13 ± 0.02, *n* = 9) (see Fig. 9*B*,*D*,*E*) showed significantly less attenuation than highly synchronous (Δ*t* = 0.1 ms: 0.05 ± 0.01, *n* = 9, *p* < 0.001) (see Fig. 9*B*,*D*,*E*) or entirely desynchronous inputs (Δ*t* = 100 ms: 0.03 ± 0.01, *n* = 9, *p* < 0.001) (see Fig. 9*D*,*E*). Across intermediate EPSC intervals, somatic EPSP amplitude showed relatively little variation (see Fig. 9*D*). This results from enhanced D→S voltage transfer of EPSPs at intermediate input frequencies because slowly rising compound EPSPs are less efficiently filtered by membrane capacitance as they propagate toward the soma compared with highly synchronous (0.1 ms) or entirely desynchronous (100 ms) EPSPs. We found similar effects in our computational model, where a clear optimum temporal integration window for inter-EPSCs intervals between 2 and 30 ms was observed (see Fig. 9*E*). Figure 9*C* illustrates the propagation of compound EPSPs (Δ*t* = 10 ms) along a dendrite in our computational model and the effect temporal summation has upon voltage attenuation.

These data indicate that, to act as efficient “modulators” (Sherman and Guillery, 1998) of TC neuron somatic membrane potential and firing rate, CT synaptic input would need to fall into this temporal integration window. Inter-EPSC intervals of 2 and 30 ms equate to input rates of 500 and 33 Hz. Given that each TC neuron receives ∼3000–4000 CT inputs (Jones, 2002), all of which have equal somatic weighting, these input rates would be physiologically plausible *in vivo*.

### Temporal input pattern-dependent corticothalamic EPSP amplification in thalamocortical neuron dendrites

**Figure 4.**
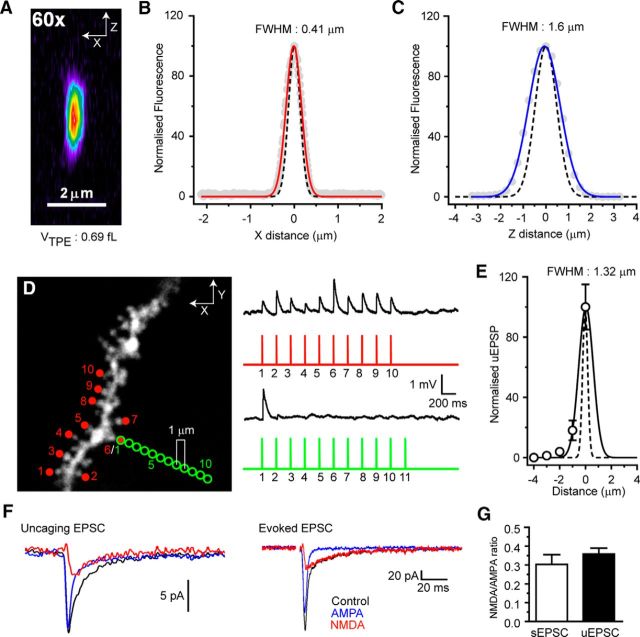
Control experiments for 2-photon glutamate uncaging. ***A***, Pseudocolor image of a 0.17 μm PS-speck fluorescent microsphere revealing the axial (X-Z) imaging (810 nm) point-spread function (PSF) of our microscope fitted with 60×, 1.0 NA objective lens (Olympus LUMPlan FL/N). ***B***, Lateral (X) PSF. Gray circles represent normalized fluorescence intensity for individual microspheres. Red line indicates average intensity. Black dashed line indicates theoretical diffraction-limited PSF (see Zipfel et al., 2003; and Materials and Methods). ***C***, Axial (Z) PSF. Gray circles represent normalized fluorescence intensity for individual microspheres. Blue line indicates average intensity. Black dashed line indicates theoretical diffraction-limited PSF. ***D***, Lateral diffusional influence of uncaged glutamate. uEPSPs evoked by glutamate uncaging onto dendritic spines of a CA1 hippocampal pyramidal neuron basal dendrite (red spots). Progressively moving the uncaging spot further (1 μm increments) from the spine head caused reduction, then loss of evoked uEPSPs (green circles). ***E***, Plot of normalized uEPSP versus distance from spine head (0 μm, maximal response). ***F***, Typical glutamate uncaging evoked EPSC from a distal TC neuron dendrite (average of 20 individual events) and a synaptically evoked EPSC at −50 mV. Black traces represent control. Blue traces represent AMPA EPSC (in 50 μm d-AP5). Red traces represent NMDA EPSC (Control-AMPA EPSC). ***G***, NMDA-to-AMPA ratio for uEPSP and synaptically evoked EPSP (*p* > 0.05, *n* = 5).

**Figure 5.**
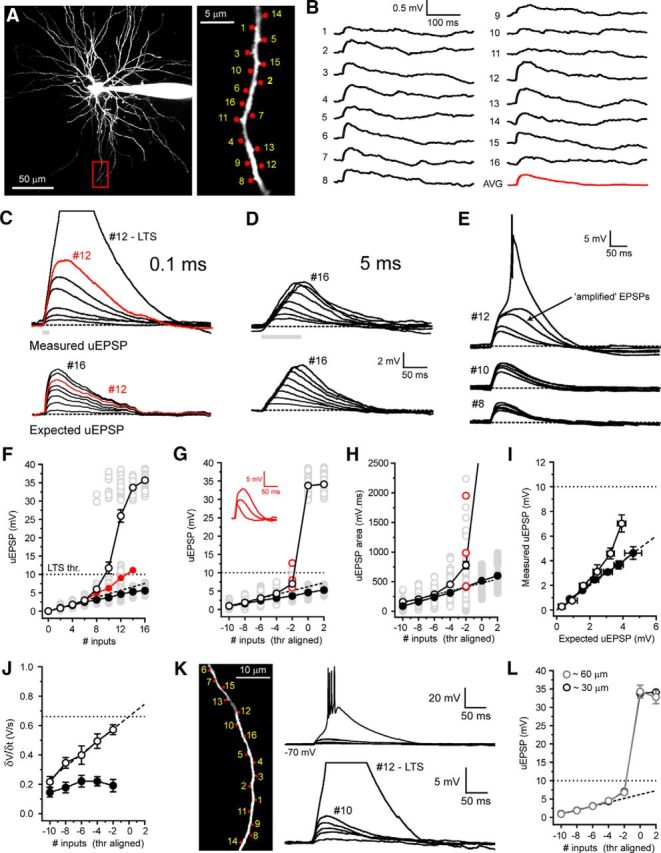
Synchrony-dependent nonlinear amplification of corticothalamic synaptic input to hyperpolarized thalamocortical neurons. ***A***, Two-photon fluorescence image of a TC neuron showing the location of glutamate uncaging spots. Area bounded by red box is shown enlarged (right). ***B***, Individual uEPSPs evoked by glutamate uncaging at the numbered spots indicated in ***A*** with 400 ms interspot intervals. ***C***, Example traces represent responses to increasing numbers of uncaging spots (0.1 ms interval) delivered to the dendrite shown in ***A***. Bottom traces represent the expected sum of the individual uEPSPs in ***B***. ***D***, Same as in ***C*** but for interspot interval of 5 ms. ***E***, Trial-to-trial variability of uEPSPs with increasing numbers of inputs (0.1 ms, #8–12) near LTS threshold. Note the occurrence of varying degrees of uEPSP amplification and LTSs with equal input numbers (#12). ***F***, uEPSP amplitude versus number of uncaging inputs delivered. Gray circles represent individual uEPSPs (0.1 ms interval, *n* = 5 trials per dendrite per input number, 11 neurons) illustrating trial-to-trial variability depicted in ***E***. Filled gray circles represent individual uEPSPs (5 ms intervals, 5 trials per dendrite per input number, 6 neurons). Black circles represent mean uEPSPs (0.1 ms). Filled red circles represent mean uEPSP excluding LTSs. Dashed black line indicates linear fit to mean uEPSPs evoked by 2–6 inputs. Filled black circles represent mean uEPSP with 5 ms interval. ***G***, LTS threshold-aligned uEPSPs versus number of inputs delivered. Same symbols as in ***F***. Red circles represent the amplified uEPSPs shown in inset. ***H***, uEPSP area versus number of inputs delivered. Same symbols as in ***F***. Red circles represent the uEPSPs shown in inset (***G***). ***I***, Measured sub-LTS threshold uEPSPs versus expected arithmetic sum of individual uEPSPs. ***J***, Maximum rate of rise (δV/δt) for sub-LTS threshold uEPSPs at 0.1 ms (black circles) and 5 ms (filled black circles) intervals. ***K***, Two-photon fluorescence image showing the distributed location of glutamate uncaging spots. Traces represent uEPSPs evoked by uncaging inputs distributed (∼60 μm) over a single dendritic branch. ***L***, Plot of the mean, threshold-aligned I-O function for inputs delivered to single dendritic branches in clustered (∼30 μm, black circles) or distributed (∼60 μm) patterns.

The previous experiments provide new insight into dendritic propagation of CT EPSPs but do not explain integration of different spatiotemporal patterns of input. Moreover, aEPSPs do not recapitulate the effects of physiological synaptic activation involving glutamate release. To overcome this problem, we used patterned 2-photon uncaging of MNI-glutamate. Unlike dendrites of other glutamatergic neurons, TC neuron dendrites lack dendritic spines, particularly at distal locations where the majority of CT input arrives (see Figs. 5*A*,*K*, 8*A*). Therefore, uEPSPs (0.5 ms uncaging time, λ = 720 nm) were produced by sequential MNI-glutamate uncaging at up to 16 spots placed close to a chosen distal dendrite (100–150 μm from soma; see Figs. 5*A*, 8*A*). Uncaging spots were separated by a minimum of 2 μm and targeted in random order to reduce the risk of coincident activation of individual synapses by more than one uncaging stimulus. In TC neurons, voltage-clamped at −50 mV, we found that the NMDA-to-AMPA ratios of uEPSCs were not significantly different from those of small EPSCs evoked by electrical stimulation of CT afferents, suggesting that uncaging did not recruit excessive levels of perisynaptic or extrasynaptic NMDA receptor activation (evoked EPSP: 0.30 ± 0.05, uEPSP: 0.36 ± 0.03, *n* = 5, *p* > 0.05) (Fig. 4*F*,*G*).

To investigate temporal integration of CT EPSPs, we measured uEPSP summation with both highly synchronous (0.1 ms move time and 0.5 ms uncaging time per spot, all 16 inputs within 9.5 ms; Fig. 5*C*, gray bar) and asynchronous (5 ms, 83 ms, Fig. 5*D*, gray bar) inputs. To begin, we evoked increasing numbers of synchronous uEPSPs at rest (−70 mV). Activating 2–6 synapses on a single distal dendritic branch (Fig. 5*A*) resulted in somatic uEPSPs whose amplitude increased linearly with increasing numbers of stimuli (*n* = 11) (Fig. 5*C*,*F*). However, increasing the number of activated synapses produced nonlinear responses taking the form of “amplified” uEPSPs that could, upon activation of sufficient numbers of synapses, readily trigger low threshold Ca^2+^ spikes (LTSs) (*n* = 11) (Fig. 5*C*,*E*,*F*). Amplified uEPSPs showed a high degree of intradendrite and intercell trial-by-trial variability as a consequence of background “noise” introduced by intrinsic and synaptic conductances. In individual dendrites, equal numbers of uncaging inputs produced wide ranging voltage responses varying from linear nonamplified EPSPs to full LTSs (Fig. 5*E*, #12). Figure 5*F* shows the individual uEPSPs from all trials (gray circles, *n* = 55, 5 trials per dendrite for 11 cells), illustrating the variability produced by activation of differing numbers of synapses. Nonetheless, by excluding trials where LTSs occurred from this dataset, a clear difference between the measured subthreshold uEPSPs (Fig. 5*F*, red circles) and the expected linear sum of individual inputs, extrapolated from a fit to the initial linear uEPSPs (#2–6, Fig. 5*F*, dashed line), was revealed. To quantify EPSP amplification, I-O functions were aligned to the threshold number of inputs required to evoke LTSs. Threshold-aligned uEPSPs confirmed subthreshold nonlinearity and showed high variability in maximal subthreshold responses (Fig. 5*G*). The EPSPs depicted in Figure 5*G* (inset) demonstrate the maximal subthreshold responses (i.e., the next input produced LTS) in individual trials from three different TC neuron dendrites (corresponding to red circles). As well as having enhanced amplitudes, amplified EPSPs also showed marked increases in duration. Therefore, to determine the full extent of EPSP amplification, we also measured uEPSP area. In line with enhanced amplitude, the area of uEPSPs evoked by synchronous inputs was also highly nonlinear (Fig. 5*H*). However, whereas the largest subthreshold uEPSPs showed only a twofold increase in maximum amplitude compared with the expected EPSP (Fig. 5*G*), as a consequence of their plateau-like nature, uEPSP area was increased by up to fourfold (Fig. 5*H*). Next, to determine whether amplified uEPSPs were truly greater than the sum of their individual inputs, in neurons where unitary uEPSPs could be well resolved (400 ms intervals, *n* = 6) (Fig. 5*B*), we compared threshold-aligned measured uEPSP amplitudes with the expected sum of single uEPSPs. Measured uEPSPs showed significant amplification compared with the expected sum of individual inputs (measured uEPSP 10 inputs: 7.02 ± 0.70 mV, expected EPSP 10 inputs: 3.92 ± 0.25 mV, *n* = 6, *p* < 0.01) (Fig. 5*C*,*I*). Importantly, in these experiments, no step-like increase in the peak temporal derivate of the somatic voltage responses was observed (Fig. 2*J*), indicating that nonlinearity represents a graded response rather than an all-or-none dendritic spike-like response. Thus, with sufficient synchronous input, CT EPSPs are greater than the simple sum of individual EPSPs. Finally, we found no difference in nonlinear EPSP amplification when inputs were distributed more widely across a single branch (maximum subthreshold EPSP 30 μm: 7.88 ± 1.78 mV, *n* = 11, 60 μm: 6.68 ± 0.80 mV, *n* = 5, *p* > 0.05) (Fig. 5*K*,*L*), suggesting that individual dendritic branches act as single integrative compartments.

**Figure 6.**
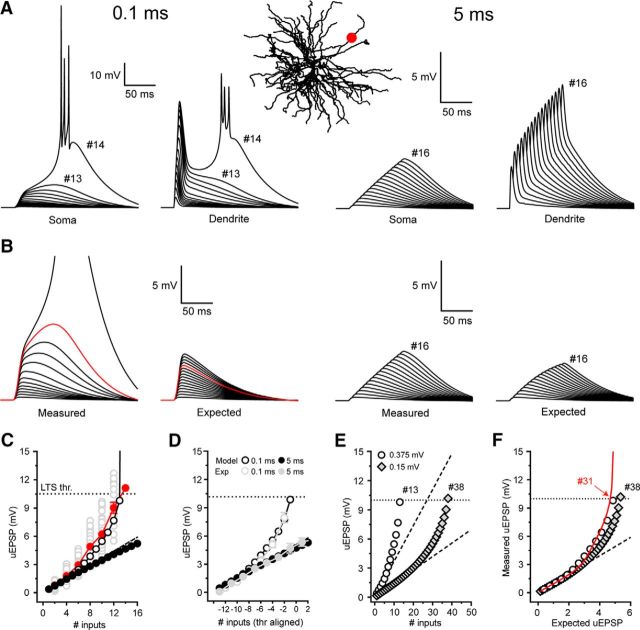
Simulated synchrony-dependent nonlinear EPSP amplification with quantal-sized CT inputs. ***A***, Simulated somatic and dendritic responses to increasing numbers of synchronous (0.1 ms) and asynchronous (5 ms) uEPSP-sized model inputs. The dendritic input and recording location is indicated by the red circle overlaid onto a 2-dimensional projection of the model TC neuron. ***B***, Measured EPSPs evoked by increasing numbers of inputs versus the expected sum of individual simulated EPSPs for each temporal input pattern. Red traces represent the maximum measured subthreshold response and the equivalent expected EPSP with the same number of inputs. ***C***, The I-O function for simulated EPSPs delivered at 0.1 ms (black circles) and 5 ms (filled black circles) intervals. The individual experimental trials (gray circles) and the mean uEPSPs, excluding LTSs (filled red circles) as described in Figure 1, are shown for comparison. ***D***, Threshold-aligned I-O function of simulated (gray) and experimental (black) EPSPs for each different temporal input pattern. ***E***, I-O function for simulated EPSPs evoked by uncaging sized inputs (black circles) and quantal-sized inputs (filled gray diamonds). Note the increased number of inputs required to evoke nonlinear responses with smaller EPSPs but equivalent voltage threshold for the nonlinearity. ***F***, Measured EPSPs versus expected EPSPs for uncaging sized (black circles) and quantal-sized (filled gray diamonds) inputs reveal similar I-O functions. Red line indicates EPSPs evoked by quantal size inputs delivered at 0.24 ms intervals to match the δV/δt obtained with uncaging sized inputs and allow all input to be delivered in the equivalent temporal window (9.5 ms). This reduces the threshold number of quantal-sized inputs to evoke LTSs from 38 to 31 inputs.

Next, we tested whether nonlinear EPSP amplification occurred in our previously described TC neuron computational model (Connelly et al., 2015). Experiments were performed by adding 16 conductance-based synapses, whose amplitudes and NMDA-to-AMPA ratios were equivalent to experimental uEPSPs, to an individual dendritic branch (Fig. 6*A*). Simulations revealed a robust, graded nonlinear increase in EPSP amplitude with activation of increasing numbers of inputs (Fig. 6*A–D*). Moreover, nonlinearity in our model, in the absence of background synaptic “noise,” was remarkably similar to the mean nonlinearity observed in uncaging experiments (Fig. 6*C*, black circles vs filled red circles). In both experiments and simulations, amplified EPSPs occurred with >6 synchronous inputs and 12–13 inputs were sufficient to reach LTS threshold. However, for technical reasons, the uEPSPs used in this study are 2–2.5 times larger than single measured quantal CT EPSPs (10.1 pA). Therefore, we tested whether quantal-sized EPSPs were capable of evoking nonlinear responses using our computer model. When model EPSPs were reduced from 0.37 mV to 0.15 mV, similar nonlinear EPSP amplification was achieved with activation of increasing numbers of synapses (Fig. 6*E*,*F*). Notably, in this case, 20 inputs were required to evoke nonlinear EPSPs and 38 inputs (vs 13 simulated uEPSP-sized inputs) were required to reach LTS threshold. By reducing the interval between simulated quantal EPSPs to 0.24 ms (from 0.5 ms), we could deliver the greater number of inputs within the same temporal window (9.5 ms) and with the same rate of membrane potential rise (δV/δt) as experimental uEPSPs. With this input pattern, plots of measured EPSPs versus the expected summed EPSPs matched those obtained with larger uEPSP-sized inputs (Fig. 6*F*) and only 31 inputs were required to reach LTS threshold. Thus, we conclude that integration of quantal-sized CT EPSPs would be capable of engaging nonlinear amplification. Intriguingly, EPSP amplification occurred between −66 and −60 mV, suggesting a voltage-dependent contribution of T-type Ca^2+^ channels to EPSP amplification (Figs. 5, Fig. 6).

In comparison, asynchronous (5 ms intervals) uncaging inputs (2–16 inputs) did not produce nonlinear amplification either *in vitro* (*n* = 6) (Fig. 5*D*,*F–H*) or in simulations (Fig. 6*B–D*). Indeed, the increase in asynchronous uEPSP amplitude with increasing numbers of inputs was initially linear and then sublinear, as would be expected for linearly summing individual EPSPs with exponential decays (Figs. 5*D*,*G*, 6*B–D*). Furthermore, unlike synchronous uEPSPs, the area of asynchronous uEPSPs also increased in a linear fashion (Fig. 5*H*). Consequently, we found that experimentally measured asynchronous uEPSPs matched the expected sums of individual uEPSPs (Fig. 5*D*,*I*). The linearity observed with asynchronous input supports the idea that amplification of synchronous input relies upon a voltage- and time-dependent mechanism. Thus, whereas asynchronous uEPSPs depolarize TC neurons to a similar degree as synchronous input (Fig. 5*D*,*F–H*), their slower rate of rise (δV/δt; Fig. 5*I*) coupled with the voltage- and time-dependent inactivation properties of T-type Ca^2+^ channels appears to preclude EPSP amplification.

**Figure 7.**
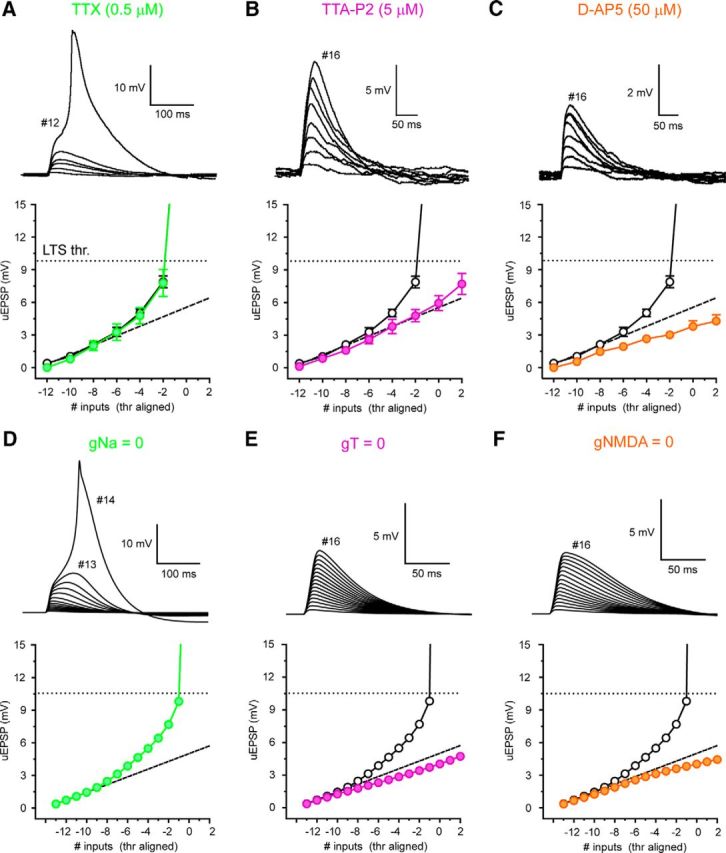
Mechanism of nonlinear corticothalamic EPSP amplification in hyperpolarized TC neurons. ***A***, Traces represent synchronous (0.1 ms) uncaging evoked EPSPs in the presence of TTX at −70 mV. Filled green circles represent threshold-aligned uEPSPs versus input number in TTX (0.5 μm). Black circles represent control. ***B***, Same as in ***A*** for TTA-P2 (filled purple circles, 5 μm). ***C***, Same as in ***A*** for d-AP5 (filled orange circles, 50 μm). ***D***, Traces represent simulated synchronous EPSPs in the absence of Na^+^ conductance. Filled green circles represent EPSPs with gNa = 0. Black circles represent control. ***E***, Same as in ***D*** for zero T-type Ca^2+^ channel conductance (gT = 0, filled purple circles). ***F***, Same as in ***D*** for zero NMDA receptor conductance (gNMDA = 0, filled orange circles).

To test whether EPSP amplification relied upon T-type Ca^2+^ channels, we manipulated the availability of key ionic conductances. First, we found no involvement of voltage-gated Na^+^ channels (VGSCs) in EPSP amplification either experimentally (TTX 0.5 μm, *n* = 6, *p* > 0.05) (Fig. 7*A*) or in simulations (gNa = 0) (Fig. 7*D*). On the other hand, when T-type Ca^2+^ channels were pharmacologically blocked *in vitro* (TTA-P2 5 μm, *n* = 6) (Fig. 7*B*) or reduced *in silico* (Fig. 7*E*), synchronous uEPSP amplification was abolished and inputs summed in a linear manner. Finally, in the presence of the NMDA receptor blocker d-AP5 (50 μm, *n* = 5) (Fig. 7*C*) or with g_NMDA_ = 0 (Fig. 4*F*), the summation of uEPSPs was markedly sublinear. These findings demonstrate that NMDA receptor activation is required to compensate for the loss of driving force encountered by spatially and temporally clustered inputs and is necessary to maintain I-O linearity, at least across the range of voltages tested.

### Synchrony-dependent corticothalamic I-O gain enhancement by NMDA receptors

**Figure 8.**
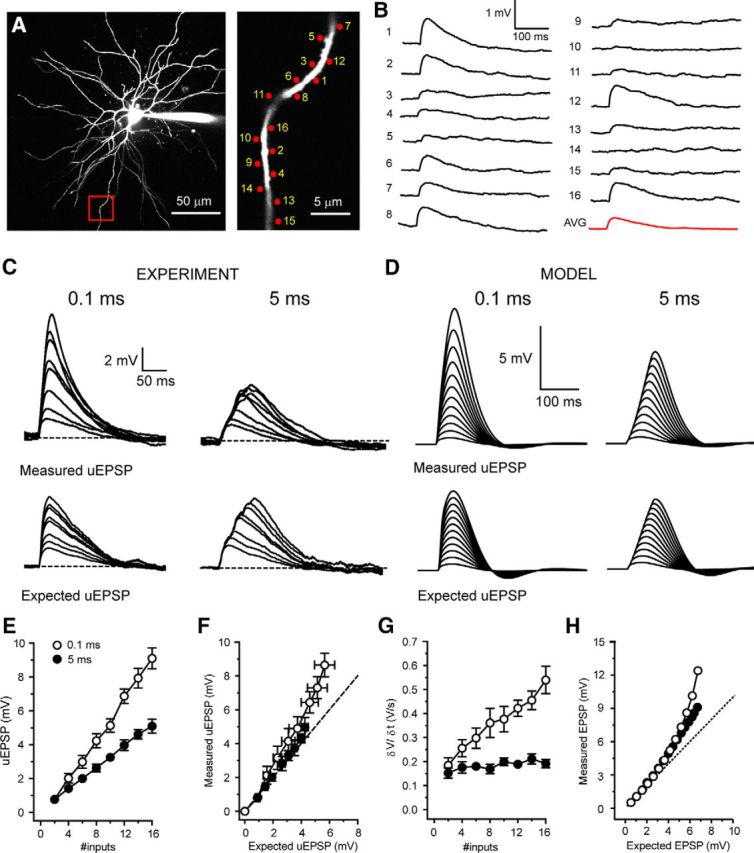
Synchrony-dependent enhanced I-O gain in depolarized thalamocortical neurons. ***A***, Two-photon fluorescence image of a TC neuron showing the location of glutamate uncaging spots. Area bounded by red box is shown enlarged (right). ***B***, Individual uEPSPs evoked by glutamate uncaging at the numbered spots indicated in ***A*** with 400 ms interspot intervals. ***C***, Example traces depicting uEPSP responses to increasing numbers of uncaging spots at 0.1 and 5 ms intervals delivered to the dendrite shown in ***A***. Bottom traces represent the expected sum of the individual uEPSPs for each temporal input pattern. ***D***, Same as in ***C*** for simulated EPSPs. ***E***, Mean uEPSPs versus number of inputs for 0.1 ms (black circles) and 5 ms (filled black circles) intervals at −55 mV. ***F***, Measured uEPSP versus expected uEPSP at −55 mV. Symbols as in ***E***. ***G***, δV/δt of uEPSPs at −55 mV versus number of inputs. Symbols as in ***E***. ***H***, Simulated measured EPSP versus expected EPSP at −55 mV. Symbols as in ***E***.

**Figure 9.**
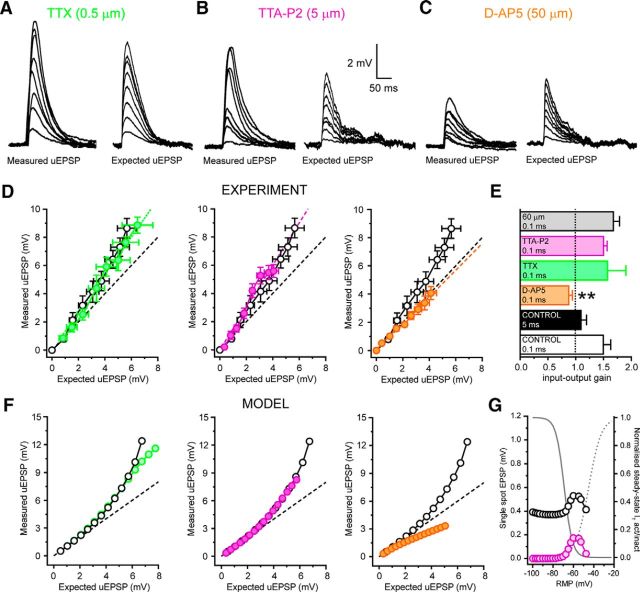
Mechanism of postsynaptic I-O gain enhancement in depolarized thalamocortical neurons. ***A***, Traces represent uEPSPs evoked by synchronous (0.1 ms) glutmate uncaging in the presence of TTX (0.5 μm) and the expected sums of individual uEPSPs from the same dendrite. ***B***, Same as in ***A*** for TTA-P2 (5 μm). ***C***, Same as in ***A*** for d-AP5 (50 μm). ***D***, Mean measured versus expected uEPSPs for synchronous inputs. Filled green circles represent TTX. Dashed green line indicates linear fit to mean uEPSPs in TTX. Filled purple circles represent TTA-P2. Dashed purple line indicates linear fit to mean uEPSPs in TTA-P2. Filled orange circles represent d-AP5. Dashed orange line indicates linear fit to mean uEPSPs in d-AP5. Black circles represent control. Dashed black line indicates unity. ***E***, Histogram summarizing I-O gain under different tested conditions. ***F***, Same as in ***D*** for simulated EPSPs at −55 mV. ***G***, Graph representing contribution of I_Twindow_ to single simulated EPSPs. Black circles represent simulated single spot EPSP amplitude at different resting membrane potentials (RMP). Purple circles represent T-type Ca^2+^ channel contribution to individual EPSPs at different resting membrane potentials. Solid gray line indicates steady-state inactivation curve for model T-type Ca^2+^ channels. Dashed gray line indicates steady-state activation curve for model T-type Ca^2+^ channels.

Having investigated CT integration in “burst-firing” mode where resting membrane potential is hyperpolarized, we next examined dendritic integration in neurons that are more depolarized, as typically occurs in “tonic-firing” mode. With activation of up to 16 spatially clustered and temporally synchronous (0.1 ms) synaptic inputs, in neurons depolarized to −55 mV, the resultant uEPSPs followed a linear I-O function (*n* = 12) (Fig. 8*C*,*E*). Nonetheless, the measured uEPSPs were notably larger than the expected algebraic sums of individual uEPSPs (measured uEPSP 16 inputs: 8.64 ± 0.70 mV, expected EPSP 16 inputs: 5.66 ± 0.71 mV, *n* = 6, *p* < 0.05) (Fig. 8*C*,*F*). Therefore, we estimated the gain of the linear I-O function (I-O gain) using linear regression and found that fits of expected versus measured uEPSPs had slopes >1 for all tested dendrites (1.05–2.03, 1.50 ± 0.13, *n* = 6) (Fig. 8*F*). Furthermore, with synchronous inputs, we saw no sudden, input-dependent increase in uEPSP amplitude or maximum δV/δt (Fig. 8*G*) characteristic of dendritic NMDA or voltage-gated Ca^2+^ spikes (Schiller et al., 2000; Augustinaite et al., 2014). When inputs to a single dendritic branch were delivered in a more spatially distributed pattern (over ∼60 μm), the I-O gain was similar to that observed with clustered input (∼30 μm: 1.48–1.87, 1.68 ± 0.11, *n* = 3, *p* > 0.05) (Fig. 9*E*). Thus, when depolarized, integration of synchronous CT input by TC neuron dendrites follows a linear I-O function, but with an I-O gain >1. This is similar to synaptic integration in hippocampal dentate gyrus granule cells (Krueppel et al., 2011).

On the other hand, as would be expected for linearly summating events whose individual decay is exponential, asynchronous uncaging inputs produced uEPSPs at −55 mV whose maximal amplitude increased in a sublinear manner (*n* = 11; Fig. 8*C*,*E*). Consequently, measured uEPSP amplitudes matched the expected sums of individual uEPSPs (measured uEPSP 16 inputs: 4.97 ± 0.58 mV, expected EPSP 16 inputs: 4.26 ± 0.24 mV, *n* = 6, *p* > 0.05) (Fig. 8*C*,*E*) and I-O gain did not deviate from unity (0.87–1.42, 1.09 ± 0.10, *n* = 6) (Fig. 8*F*).

Because T-type Ca^2+^ channels are almost entirely inactivated at −55 mV, we expected little influence of these channels on I-O gain. Indeed, in both experiments (TTA-P2 5 μm, 1.4–1.6, 1.50 ± 0.06, *n* = 3, *p* > 0.05) (Fig. 9*B*,*D*,*E*) and simulations (g_T_ = 0) (Fig. 9*F*), T-type Ca^2+^ channel removal did not affect I-O gain compared with control. Interestingly, although I-O gain was not reduced, in the absence of T-type Ca^2+^ channels, the amplitude of summed uEPSPs was decreased overall (Fig. 9*B*,*D*,*F*). Computer simulations suggest that this is due to the presence of a T-type Ca^2+^ channel “window” current in TC neurons (I_Twindow_) (Hughes et al., 1999; Dreyfus et al., 2010). At −55 mV, this small noninactivated fraction of T-type Ca^2+^ current (∼5% of maximum T-current) is sufficient to contribute to individual EPSPs and has a linear, additive effect on summed EPSPs (Deleuze et al., 2012). However, the presence of I_Twindow_ alone is not sufficient to produce nonlinear EPSP amplification similar to that observed at hyperpolarised potentials, where a significantly greater fraction of channels (∼50% at −70 mV) are available for activation (Fig. 9*G*). As with T-type Ca^2+^ channels, I-O gain was not reduced compared with control by removal of VGSCs (TTX 0.5 μm, 0.88–2.64, 1.57 ± 0.33, *n* = 5, *p* > 0.05; gNa = 0) (Fig. 9*A*,*D–F*). When VGSCs were blocked, no reduction in uEPSP size was observed, indicating that persistent Na^+^ currents do not contribute to EPSPs in a similar fashion to window T-current at this membrane potential. However, in the presence of the NMDA receptor blocker d-AP5 (50 μm) or with g_NMDA_ set to zero, EPSP amplitudes were markedly reduced (Fig. 9*C*). Moreover, I-O gain was significantly reduced compared with control in both experiments (0.70–1.035, 0.87 ± 0.07, *n* = 5, *p* < 0.01) (Fig. 6*C–E*) and simulations (Fig. 9*F*) without NMDA receptors. Indeed, in the absence of NMDA receptors, I-O gain was <1, demonstrating sublinear summation of individual EPSPs.

Thus, asynchronous CT input, as is expected to be more commonly observed *in vivo*, is integrated linearly across different membrane potentials. However, if input arrives with sufficient temporal synchrony, EPSPs are amplified by activation of T-type Ca^2+^ channels when neurons are hyperpolarized (−70 mV) and by NMDA receptor-dependent gain enhancement when depolarized (−55 mV).

**Figure 10.**
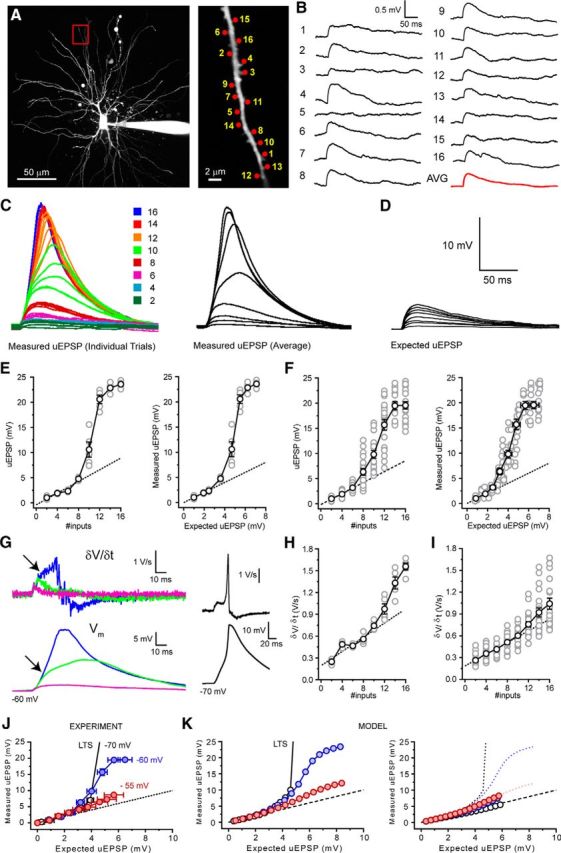
Overlapping EPSP amplification mechanisms enhance the voltage response range of thalamocortical neurons. ***A***, Two-photon fluorescence image of a TC neuron showing the location of glutamate uncaging spots. Area bounded by red box is shown enlarged (right). ***B***, Individual uEPSPs evoked by glutamate uncaging at the numbered spots indicated in ***A*** with 400 ms interspot intervals. ***C***, Example traces represent uEPSP from individual trials (5 trials per input number) evoked by delivering increasing numbers of uncaging spots to the dendrite depicted in ***A*** at 0.1 ms intervals. Traces are color coded for the number of inputs delivered as shown inset. Black traces represent average uEPSP evoked by each number of inputs. ***D***, Expected sum of individual inputs shown in ***B***. ***E***, uEPSP amplitude versus number of inputs delivered and the measured versus expected uEPSPs for the dendrite shown in ***A***. Gray circles represent individual trial uEPSPs. Black circles represent mean uEPSP. Dashed black line indicates unity. ***F***, Same as in ***E*** for all trials in every neuron tested. ***G***, Voltage responses in the cell shown in ***A*** evoked by 6, 10, and 16 uncaging inputs and their first temporal derivatives (δV/δt). A clear inflection in the δV/δt and voltage traces indicates the initiation of a “weak” LTS by the maximum number of inputs (as indicated by arrows). For comparison, the δV/δt for a full LTS evoked by 16 inputs at −70 mV (in TTX) is shown (black traces). ***H***, δV/δt versus number of inputs for the dendrite shown in ***A***. Same symbols as in ***E***. Dashed black line indicates linear fit to uEPSPs evoked by 2–6 inputs. ***I***, δV/δt versus number of inputs for all trials in every neuron tested. Same symbols as in ***E***. A step-like nonlinear increase in δV/δt is observed with 12–16 uncaging inputs as a consequence of “weak” LTS initiation. ***J***, Experimental data showing measured versus expected uEPSPs at −70 mV (black circles), −60 mV (filled blue circles), and −55 mV (filled red circles). Dashed black line indicates unity. ***K***, Left, Control simulated measured versus expected EPSPs at the membrane potentials described for the experimental data shown in ***J***. Same symbols as in ***J***. Right, Simulated measured versus expected EPSPs in the absence of T-type Ca^2+^ channels. Circles represent EPSPs (gT = 0). Dotted lines indicate EPSPs control. Same colors as in ***J***.

Nonetheless, TC neurons are not constrained to these dichotomous membrane potential states. When neurons were held at −60 mV, small numbers of inputs summed linearly with little trial-by-trial variability (Fig. 10*C*,*E*,*F*) producing uEPSPs that matched the expected sum of individual inputs (measured uEPSP 6 inputs: 3.18 ± 0.38 mV, expected uEPSP 6 inputs: 2.51 ± 0.29 mV, *n* = 4, *p* > 0.05) (Fig. 10*D–F*). However, increasing the number of inputs delivered beyond this threshold level (∼3 mV from rest) resulted in nonlinear EPSP amplification and increased trial-by-trial variability (Fig. 10*C*,*E*,*F*). For example, equal numbers of uncaging inputs (10 inputs, green traces; Fig. 10*C*) produced responses ranging from weakly enhanced EPSPs to strongly amplified plateau-like EPSPs across different trials (5 trials per input number). The I-O function at −60 mV was sigmoidal with a maximum response being achieved after activation of 12–16 inputs across different neurons (Fig. 10*E*,*F*). Maximal responses displayed spike-like characteristics with clear inflections observed in both the voltage transients (Fig. 10*C*) and their first temporal derivatives (δV/δt) (Fig. 10*G*,*H*). Because these spike-like responses at −60 mV have smaller amplitudes and δV/δt_max_ compared with LTSs evoked at −70 mV (Fig. 10*G*), we consider them to be “weak” LTSs. Comparison of maximum δV/δt (δV/δt_max_) versus the number of activated inputs at −60 and −70 mV reveals a clear threshold level at which both “weak” and full LTSs occur (∼0.7 V/s) (Figs. 5*H*, 10*H*,*I*). Our computational model accurately reproduced the I-O function that we observed experimentally at −60 mV (Fig. 10*J*,*K*). Unlike at −55 mV, where T-type Ca^2+^ channels do not contribute to increased I-O gain, when we removed these channels from simulations at −60 mV, a marked reduction in nonlinearity was revealed (Fig. 10*K*). However, at −60 mV, even in the absence of T-type Ca^2+^ channels, the nonlinear response was not completely abolished; a clear difference to the amplification found at more hyperpolarized membrane potentials (Figs. 7*B*,*E*, 10*K*). We found that the residual nonlinear component of the response at −60 mV was reliant upon NMDA receptor activation. Thus, due to the overlapping voltage dependence of T-type Ca^2+^ channels and NMDA receptors, EPSP amplification mechanisms in TC neurons do not operate independently of each other but can cooperate to amplify synchronous CT EPSPs.

### Spatially distributed corticothalamic input activates EPSP amplification mechanisms

Until now, our experiments have investigated spatially clustered synaptic input delivered across 30–60 μm of a single dendritic branch. Although such patterns are feasible, it is more likely, considering the number of synapses across the whole dendritic tree versus those on a single branch, that TC neurons receive temporally coincident inputs from cortex that are widely distributed in space. However, testing three dimensionally distributed input patterns on stellate-shaped neurons *in vitro* is at the limit of current scanning and uncaging technology. Therefore, we used our computational model (Connelly et al., 2015) to simulate how CT inputs distributed across the dendritic tree are integrated.

**Figure 11.**
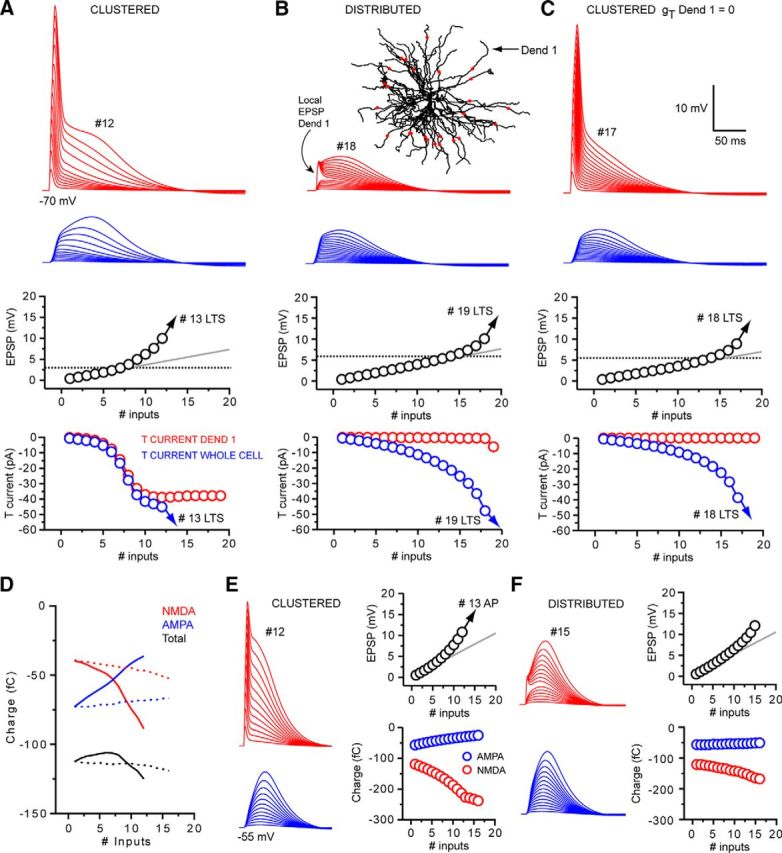
Spatially distributed synchronous corticothalamic EPSPs are amplified by T-type Ca^2+^ channel and NMDA receptor-dependent mechanisms. ***A***, Dendritic (red) and somatic (blue) EPSPs generated by increasing numbers of spatially clustered inputs onto a distal dendrite of our computational model at V_m_: −70 mV (Dend 1 in ***B***). EPSPs are amplified in a nonlinear manner before reaching LTS threshold at 13 inputs (#13 LTS). In the clustered input case, nonlinear EPSP amplification is due to the local dendritic recruitment of T-type Ca^2+^ channels by large dendritic EPSPs. Below LTS threshold, the T-type Ca^2+^ current generated locally in Dend 1 represents the majority of the T-type Ca^2+^ current in the whole cell. ***B***, Somatic and dendritic EPSPs evoked by spatially distributed synaptic input. Red dots superimposed onto the image of our model cell represent the input locations for the distributed synaptic input. Spatial input patterns were randomly generated, but each input site was used only once per input sequence (the number of possible input locations is greater than the number of inputs delivered). The larger local dendritic EPSP with input into Dend 1 where the dendritic voltage was recorded. In the distributed input case, 19 inputs were required to produce an LTS compared with 13 in the clustered case. Because of the smaller dendritic depolarization, T-type Ca^2+^ current currents in individual dendrites are markedly reduced with distributed input. ***C***, Traces represent the somatic and dendritic response to the clustered input pattern after removal of T-type Ca^2+^ channels from Dend 1 only. In the absence of dendritic T-type Ca^2+^ channels in the stimulated dendrite, EPSP summation and amplification are almost identical to the distributed input case. ***D***, Differential contribution of AMPA and NMDA receptors to EPSP with clustered and distributed input. Blue lines indicate charge input to the neuron via AMPA receptors. Red lines indicate charge through NMDA receptors. Black lines indicate total charge for increasing numbers of inputs. Solid lines indicate the clustered input case. Dashed lines indicate distributed input. ***E***, Somatic and dendritic responses to clustered synaptic input at −55 mV. Enhanced I-O gain occurs as a consequence of markedly increasing contribution of NMDA receptors with increasing input numbers. ***F***, Responses to dendritically distributed inputs at −55 mV. I-O gain is similar to the clustered case despite a much smaller increase in NMDA receptor charge with increasing inputs due to the relative lack of decrease in AMPA receptor-mediated component.

To compare spatially distributed inputs versus clustered inputs, 25 input locations were selected on distal model dendrites (Fig. 11*B*, red dots), and up to 16 of the 25 potential synapses were randomly activated (each synapse only being activated once per stimulus pattern). In comparison, for clustered patterns, all input was delivered to a single dendrite (Dend 1; Fig. 11*B*). When synapses were activated at −70 mV, the I-O curve for spatially distributed EPSPs was right shifted compared with clustered inputs (Fig. 11*A*,*B*). This increased the voltage threshold at which nonlinear EPSP amplification occurred and reduced the extent of subthreshold EPSP amplification (Fig. 11*A*,*B*). Consequently, a greater number of synchronous events were required to produce EPSP amplification or LTSs with distributed input (19 EPSPs) versus clustered input (13 EPSPs). This led us to ask why clustered input can produce greater EPSP amplification with fewer inputs than distributed synapses.

Our model reveals that high dendritic input resistance (Connelly et al., 2015) and nonlinear NMDA receptor activation allows spatially clustered, synchronous inputs to produce large local dendritic depolarizations (Fig. 11*A*). Once above threshold, these EPSPs recruit local “within-branch” dendritic T-type Ca^2+^ channels as they propagate toward the soma-producing EPSPs that are larger than the expected sum of individual inputs (Fig. 11*A*). As such, we found that when T-type Ca^2+^ channels are removed from only a single dendrite (Fig. 11*C*,*D*), the I-O function obtained by clustered stimulation of that dendrite becomes remarkably similar to that observed with spatially distributed input (Fig. 11*B*). These findings suggest that, whereas global depolarization is required to activate sufficient T-current to evoke LTSs (Connelly et al., 2015), clustered synaptic activation of a single dendritic branch can recruit enough “within-branch” T-current to amplify CT synaptic potentials. This further supports the notion that distal TC neuron dendritic branches can operate as individual integrative compartments. In contrast, summation of local dendritic EPSPs with EPSPs generated by synapses distributed across the dendritic tree results in much smaller local voltage changes that are not large enough to recruit substantial “within-branch” dendritic T-current (Fig. 11*B*). Thus, although both clustered and distributed inputs produce EPSP amplification, clustered synapses can do so with fewer coincident inputs by recruiting significant “within branch” T-current in the stimulated dendrite.

Although distributed input is less effective in evoking nonlinear signaling in TC dendrites, it is important to consider this finding next to the total number of inputs available. Whereas 19 distributed inputs (or 56 quantal-sized inputs) were required to reach LTS threshold, compared with 13 clustered inputs (38 quantal-sized), the total number of CT synaptic inputs distributed across the entire dendritic tree is ∼3000–4000, compared with perhaps only 100–300 on an individual dendritic branch. Because, as a consequence of passive normalization, all CT inputs have the same weighting at the soma, our data predict that temporally synchronous activation of as few as 1.4%–1.9% of distributed CT synapses could evoke LTSs.

The I-O function for temporally synchronous EPSPs at −55 mV is linear but with gain >1. At this membrane potential, simulated distributed input produced an I-O function similar to clustered input but with a slightly reduced I-O gain, requiring 16 inputs (Fig. 11*F*) to reach action potential threshold compared with 13 (Fig. 11*E*). In the clustered case, increased I-O gain results from the enhanced activation of NMDA receptors by large local dendritic EPSPs. On the other hand, when inputs are distributed in space, such large local dendritic EPSPs do not occur and the amplitude of summed inputs is much smaller (Fig. 11*F*). Therefore, we examined how, in the absence of large local dendritic depolarization, distributed inputs produce I-O gain not dissimilar to clustered inputs. By comparing charge delivered to the cell by NMDA receptors versus that arriving through AMPA receptors during clustered input sequences, we found that NMDA receptor-dependent charge transfer increased significantly as the number of inputs increased (#1: 119.2 fC, #12: 212.7 fC) (Fig. 11*D*,*E*). Conversely, recruitment of NMDA receptors was much less with increasing numbers of distributed inputs (#1: 121.4 fC, # 15: 164.9 fC) (Fig. 11*D*,*F*). Nonetheless, whereas NMDA-mediated charge transfer is greater in the clustered case versus the distributed case, when input is spatially clustered, the resulting large dendritic EPSPs reduce local driving force and, consequently, the charge contributed by AMPA receptors (Fig. 11*E*). In contrast, because local dendritic EPSPs are relatively small, distributed inputs do not suffer from a noticeable reduction in driving force and AMPA-mediated charge transfer remains nearly constant as the number of inputs increases (Fig. 11*F*). Thus, in the clustered case, because increased NMDA receptor-mediated charge must compensate for a reduced AMPA contribution, the total charge transfer is only slightly greater than when inputs are distributed (total charge clustered #16: 263.5 fC, distributed #16: 218.6). This mechanism minimizes the difference in I-O gain between spatially clustered and distributed synaptic input. Thus, both clustered and distributed CT inputs can be amplified by NMDA receptor and T-type Ca^2+^ channel-dependent mechanisms, providing they are sufficiently temporally coincident.

**Figure 12.**
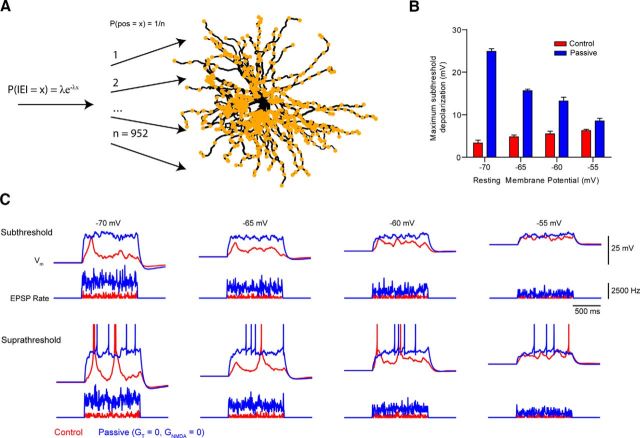
Nonlinear mechanisms limit the magnitude of subthreshold membrane potential modulation. ***A***, Schematic representation of the computer simulation. Timing of EPSPs was determined by sampling from a Poisson distribution, and the spatial location of the synaptic input was chosen randomly from a set of inputs on the distal dendrites. Simulations were run repeatedly over a range of mean interevent intervals, and the rate of input was increased until the cell spiked on >50% of trials. Simulations were run at different resting potentials, and in the presence or absence of nonlinear mechanisms (NMDA receptors and T-type Ca^2+^ channels). During the simulation run, the membrane potential during the last 500 ms of the input train was averaged. ***B***, The result of the simulation showing that across a range of membrane potentials, the maximum subthreshold depolarization reached during input trains was significantly higher when nonlinear mechanisms were removed (*p* < 0.0001). ***C***, Example waveforms showing the voltage response of neurons at different membrane potentials to random trains of input at frequencies just below that needed to cause spiking (subthreshold), and just above it (suprathreshold). Action potentials are truncated for clarity.

Intriguingly, our data suggest that such input amplification might limit the ability of CT inputs to act solely as modulators of membrane potential. As such, when input is asynchronous, exponentially decaying unitary EPSPs add together to produce summed EPSPs whose maximal amplitudes increase in a sublinear fashion. Consequently, asynchronous EPSPs are unable to produce significant neuronal depolarization. On the other hand, when input is sufficiently synchronous, it is amplified and can behave more like a “driver” input. To test this theory, simulated, independent CT synapses, located across all distal dendritic segments, were spatially and temporally activated at random using 1-s-long Poisson input trains (Fig. 12*A*) (see Materials and Methods). The mean frequency of input was then progressively increased until spikes were observed during >50% of input trains (*n* = 5 trials per input frequency). We found that, at a resting membrane potential of −70 mV, a mean steady-state depolarization of only 3.3 ± 0.7 mV could be achieved before the input frequency was sufficient to drive LTSs (Fig. 12*B*,*C*). However, when NMDA receptors and T-type Ca^2+^ channels were blocked, significantly higher frequencies were required before the summed inputs could produce depolarization (24.9 ± 0.6 mV) capable of driving spikes (Fig. 12*B*,*C*). Similarly, at −65, −60, and −55 mV, simulated CT input was able to depolarize cells only by 4.8 ± 0.4, 5.5 ± 0.6, and 6.3 ± 0.3 mV, respectively, before spikes were generated. In the passive case, much higher frequency inputs could achieve depolarization of 15.6 ± 0.4, 13.2 ± 0.9, and 8.5 ± 0.6 mV without driving spiking (Fig. 12*B*,*C*). As such, nonlinear EPSP amplification mechanisms can constrain the ability of CT inputs to act as membrane potential “modulators” to only a few millivolts above rest. Nonetheless, this degree of depolarization of TC neurons by CT feedback has recently been shown to be sufficient to have significant effects on relay efficiency and spike-output mode *in vivo* (Mease et al., 2014).

## Discussion

Corticothalamic feedback to TC neurons is extensive, playing a critical role in thalamic function (Sillito and Jones, 2002; Briggs and Usrey, 2008). Here, we find *in vitro* that TC neuron dendritic electrical properties cause passive normalization of CT EPSP amplitude. This effect means that all CT synapses, regardless of their physical input location, have equal weighting and influence on the somatic membrane potential (Fig. 10*A*). In addition, we demonstrate that temporally asynchronous CT inputs are integrated in a linear manner, but synchronous EPSPs are amplified by T-type Ca^2+^ channels and NMDA receptors.

### Passive normalization of CT synaptic input in TC neurons

Passive cable theory predicts the ability of a synapse to alter somatic membrane potential declines as its distance from the soma increases (Rall, 1970). However, this is not true for all dendrites (Jaffe and Carnevale, 1999) and in TC neurons, CT-EPSPs are powerfully normalized, resulting in somatic amplitudes that are virtually independent of dendritic input location. This synaptic normalization is passive and does not rely on scaling of synaptic conductances as occurs in hippocampal CA1 pyramidal neurons (Magee and Cook, 2000; Smith et al., 2003) because the distance-dependent amplitude distribution and dendrite to soma voltage transfer of CT sEPSPs were nearly identical to those of aEPSPs evoked by injection of equal-sized aEPSCs throughout the dendritic tree.

How do the membrane properties of TC neurons produce the conditions under which passive normalization occurs? In simple terms, the soma of TC neurons act as a powerful current sink; and this, coupled with resistive dendrites, forces nearly all current injected by individual CT synapses to flow to the soma. Thus, CT synapses act like current sources rather than voltage sources; and as such, the somatic EPSP is largely determined by local somatic input resistance and capacitance (Jaffe and Carnevale, 1999).

Although it remains to be demonstrated *in vivo*, passive normalization might allow TC neurons to “democratically” sample ongoing activity of large layer VI pyramidal neuron ensembles by equalizing the weighting of several thousand spatially distributed CT synaptic inputs. Indeed, because passive normalization occurs independently of resting membrane potential and is insensitive to increases in membrane conductance resulting from sustained synaptic bombardment *in vivo* (Jaffe and Carnevale, 1999), this mechanism could also allow TC neurons to receive a consistent and reliable “report” of behavioral state-dependent cortical output during highly active network conditions. Such a mechanism, therefore, might allow top-down CT feedback to rapidly signal the relevance of incoming sensory information to TC neurons and by depolarizing their membrane potential enhance their relay efficiency (Mease et al., 2014).

### Spatial and temporal integration of CT input in TC neurons

dLGN TC neurons receive two major sources of glutamatergic input: sensory inputs from retinal ganglion cells and feedback inputs from cortical layer VI neurons (Wilson et al., 1984; Liu et al., 1995; Sherman and Guillery, 1996; Jones, 2009). Retinal ganglion cell axons form large terminals on proximal dendrites and produce unitary EPSPs that drive TC neuron spiking to “relay” visual information to cortex (Sherman and Guillery, 1996; Jones, 2009). Conversely, CT synaptic terminals are smaller, found on distal regions of the dendritic tree and produce unitary EPSPs that, in isolation, cannot drive spiking (Wilson et al., 1984; Van Horn et al., 2000; Jones, 2002). These facts have resulted in the idea that retinal ganglion cell inputs are “drivers” of TC neuron firing whereas CT inputs act as “modulators” of TC neuron activity (Sherman and Guillery, 1998; Sherman, 2007). Our data reveal that activation of as few as 30 CT inputs, arriving on an individual distal dendritic branch within a sufficiently narrow time window, can activate a nonlinear mode of synaptic integration. This active CT integration mode is mediated by postsynaptic mechanisms involving NMDA receptors and T-type Ca^2+^ channels and acts to amplify cortical feedback to TC neurons. Thus, as well as acting as “modulators” of TC neuron activity, our data suggest that CT synapses could also act as conditional “synchrony-dependent” drivers of thalamic firing.

Despite this, it remains clear that TC neuron spikes are mostly evoked by sensory “driver” EPSPs and that *in vivo* CT-driven spikes are rare. This is because the addition of nonsensory spikes would likely be detrimental to information transfer fidelity (Cudeiro and Sillito, 2006). Nonetheless, our new findings suggest that synchronous CT activity triggered by pertinent incoming sensory signals could evoke large subthreshold T-type Ca^2+^ channel and NMDA receptor-dependent (50–100 ms) plateau-like EPSPs in TC neurons (Figs. 2*C*,*E*, 3*A*,*B*, 7). These long-lasting EPSPs would depolarize the entire somatodendritic tree (Connelly et al., 2015), transiently opening a temporal window during which sensory EPSPs could more easily reach spike threshold and be relayed to cortex. This feedback mechanism is plausible because, in macaque monkeys, a class of layer VI CT neurons have been identified that not only receive direct input from thalamus but also send fast-conducting (1–7 ms) feedback axons back to thalamus, allowing CT feedback to impact on signaling in the dLGN within 30–50 ms of the presentation of a visual stimulus (Briggs and Usrey, 2007).

As well as projections to TC neurons, axons of CT neurons send collateral projections to inhibitory neurons of the TRN, which, in turn, project to thalamic nuclei to produce GABAergic inhibition (Sherman and Guillery, 1996; Jones, 2002). Based on previous studies (Turner and Salt, 1998; Von Krosigk et al., 1999; Cruikshank et al., 2010; Jurgens et al., 2012), it is typically thought that CT output produces disynaptic inhibition of TC neurons and that indirect GABAergic input through the CT-TRN-TC pathway dominates over direct CT-TC excitation. Several recent studies call this view into question, suggesting far greater spatial and temporal complexity in CT-TRN-TC circuits (Zikopoulos and Barbas, 2007; Halassa et al., 2014; Mease et al., 2014; Wimmer et al., 2015). First, at the single-cell level, the precise connectivity of feedforward CT-TRN-TC circuits remains unclear. Whereas individual CT axons connect to both TC and TRN neurons, the majority of TRN neurons do not project to TC cells that share common CT input (i.e., closed-loop CT-TRN-TC circuits) (Pinault, 2004). Instead, TRN neurons typically project to TC cells receiving input from different CT neurons to form open-loop CT-TRN-TC circuits. For closed-loop circuits, activation of CT neuron ensembles would result in spatially uniform inhibition of TC neurons, depending on whether the integrated CT input to the TRN neurons was sufficient to drive spike output (see below). On the other hand, open-loop circuits would be ideally suited to provide lateral inhibition to TC neurons. This mechanism could allow CT feedback to dynamically filter sensory information and shape the center-surround receptive fields of dLGN neurons by exciting “on-center” cells while inhibiting neighboring “off-center” cells. Indeed, in the absence of cortical feedback, dLGN neurons display reduced responsiveness to stimuli restricted to the classical receptive field (Przybyszewski et al., 2000, Jones et al., 2012), whereas stimuli extending into the extraclassical receptive field produce reduced surround suppression (Murphy et al., 1987; Sillito et al., 1993; Jones et al., 2000, 2012; Webb et al., 2002).

As well as spatial effects, produced by microcircuit configuration, timing of excitation and inhibition in CT-TRN-TC circuits, relative synaptic integrative properties of TC/TRN neurons, and the strength of each synapse will influence the response of TC neurons to CT feedback. For example, because CT inhibition is indirect (e.g., CT-TRN-TC) whereas excitation is direct, a lag of up to 10 ms is typically observed between the onset of each after cortical stimulation. *In vivo* this could allow sufficient time for TC neurons to integrate synchronous direct CT excitation and trigger amplified EPSPs before inhibition can produce a strong effect. Furthermore, although unitary CT inputs to TRN neurons are thought to be 3–4 times larger than TC neurons (Golshani et al., 2001; Jones, 2002), how they are integrated by TRN neurons remains unknown. It is clear, however, that to exert an inhibitory effect on TC neurons CT input must be sufficiently strong to make TRN neurons fire and release GABA. On the other hand, subthreshold excitatory CT input could markedly affect excitability and relay efficiency of TC neurons; and due to postsynaptic EPSP amplification, relatively few activated synapses may be required. Finally, because CT-TC synapses show strong use-dependent facilitation (Turner and Salt, 1998; Cruikshank et al., 2010; Jurgens et al., 2012) whereas TRN-TC synapses strongly depress (Von Krosigk et al., 1999; Jurgens et al., 2012), the duration and intensity of activity in CT circuits are likely to have a key role in determining how CT feedback effects TC neuron signaling (Mease et al., 2014).

In conclusion, our findings shed light on the spatiotemporal integration of excitatory CT synaptic input by TC neurons and provide further evidence that the feedback from cortex to thalamus is able to exert a range of complex responses in these cells. To fully appreciate the function of this pathway, further understanding of the integrative properties of neurons (i.e., TRN) in these circuits and a more detailed and comprehensive connectomic map of their connections must be explored.
